# Supplementary material for: EGCG-Derivative G28 Shows High Efficacy Inhibiting the Mammosphere-Forming Capacity of Sensitive and Resistant TNBC Models
Source: Molecules. 2019 Mar 15;24(6):1027. doi: 10.3390/molecules24061027 (PMC6471537; doi:10.3390/molecules24061027)
Supplement: Supplementary file 1 [file molecules-24-01027-s001.pdf]

Article

EGCG-derivative G28 shows high efficacy in reducing Breast Cancer Stem Cells-enriched population in TNBC models of acquired drug resistance

Ariadna Giró-Perafita[1,2,†], Marc Rabionet[2,3,†], Marta Planas[4], Lidia Feliu[4], Joaquim Ciurana[3], Santiago Ruiz-Martínez[2,*] and Teresa Puig[2,*]

[1] Perlmutter Cancer Center, NYU School of Medicine, 522 First Avenue, Smilow Research Building, Room 1104, New York, NY 10016; ariadna.giroperafita@nyulangone.org (A.G-P.)

[2] New Therapeutic Targets Laboratory (TargetsLab) - Oncology Unit, Department of Medical Sciences, Faculty of Medicine, University of Girona, Emili Grahit 77, 17003 Girona, Spain; m.rabionet@udg.edu (M.R.), santiago.ruiz@udg.edu (S.R-M.), teresa.puig@udg.edu (T.P.)

[3] Product, Process and Production Engineering Research Group (GREP), Department of Mechanical Engineering and Industrial Construction, University of Girona, Maria Aurèlia Capmany 61, 17003 Girona, Spain; quim.ciurana@udg.edu (J.C.)

[4] Laboratori d'Innovació en Processos i Productes de Síntesi Orgànica (LIPPSO), Department of Chemistry, University of Girona, Maria Aurèlia Capmany 69, 17003 Girona, Spain; marta.planas@udg.edu (M.P.), lidia.feliu@udg.edu (L.F.)

† These authors contributed equally to this work.

* Correspondence: santiago.ruiz@udg.edu (S.R-M.), Tel.+34-972-419-548: ; teresa.puig@udg.edu (T.P.), Tel.: +34-972-419-628

Supplementary Figure 1

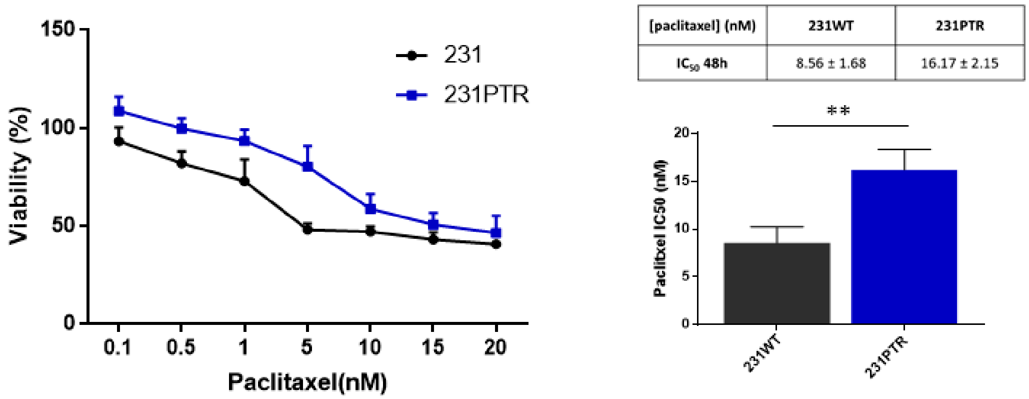

Supplementary Figure 1. Viability assays for 231 and 231PTR at increasing doses of paclitaxel for 48h. Experiments were performed at least three times in duplicate. *($p < 0.05$), **($p < 0.01$) and ***($p < 0.001$) indicate levels of statistically significance.

Supplementary Figure 2

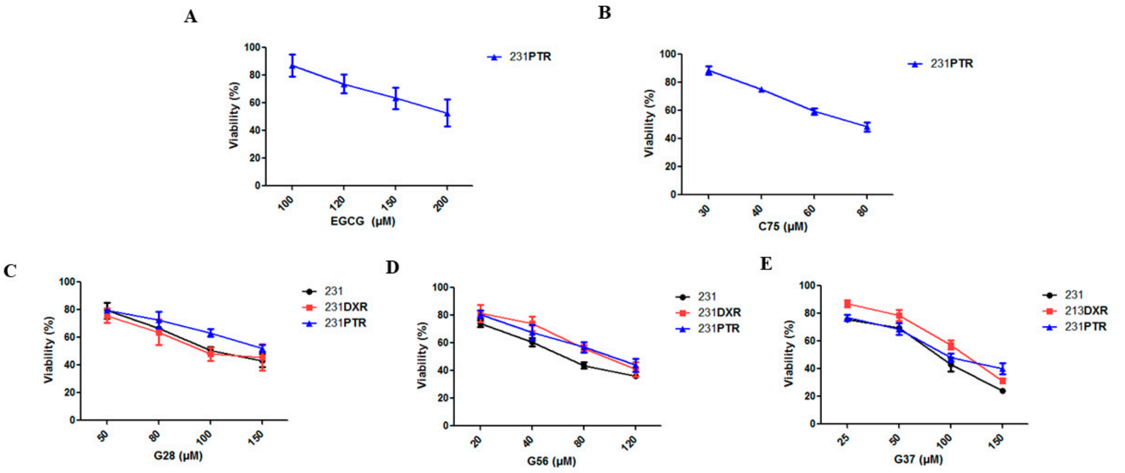

Supplementary Figure 2. Viability assays for 231, 231DXR, and 231PTR at increasing doses of FASN inhibitors for 48h. Different concentrations of (**A**) EGCG, (**B**) C75, (**C**) G28, (**D**) G56, and (**E**) G37 were tested in 231 sensitive model and 231**DXR** and 231**PTR** chemoresistant cell lines. Experiments were performed at least three times in duplicate. *($p < 0.05$), **($p < 0.01$) and ***($p < 0.001$) indicate levels of statistically significance.

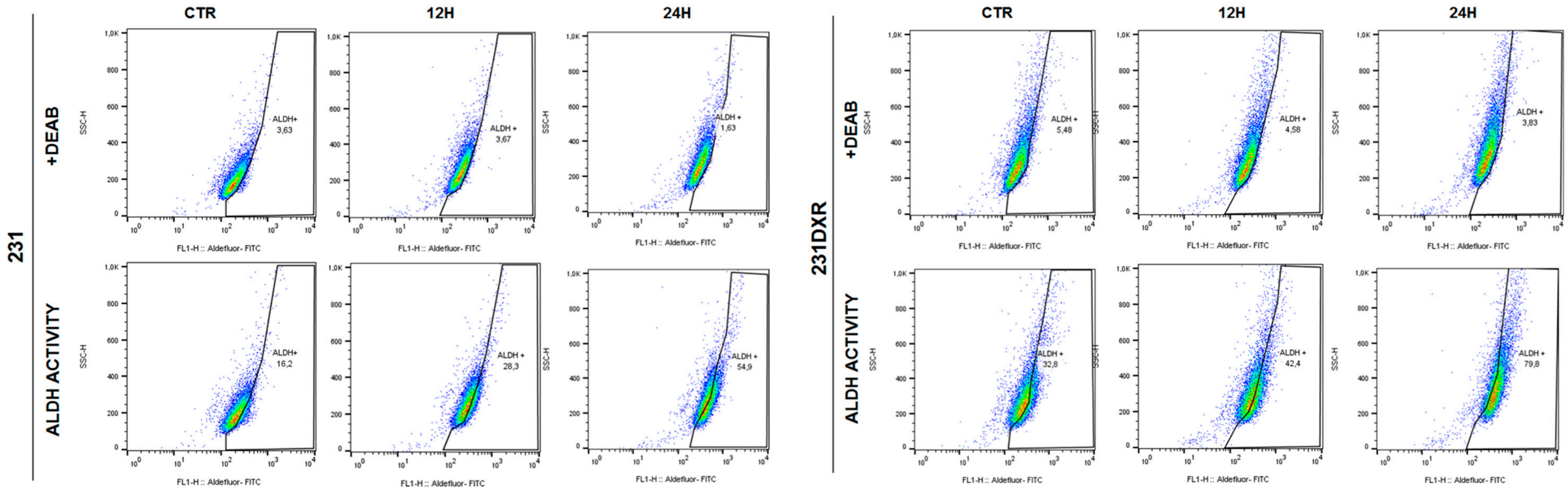

Supplementary Figure 3. ALDEFLUOR plots for 231 (left part) and 231DXR (right part) cells after doxorubicin treatment. Gates from samples with ALDH1 inhibitor DEAB (+DEAB; upper panel) and ALDH reagent (ALDH activity, lower panel) are presented. These are representative results from at least three independent experiments.
